# Seasonal Variation of the Canopy Structure Parameters and Its Correlation with Yield-Related Traits in Sugarcane

**DOI:** 10.1155/2013/801486

**Published:** 2013-12-26

**Authors:** Jun Luo, Youxiong Que, Hua Zhang, Liping Xu

**Affiliations:** Key Laboratory of Sugarcane Biology and Genetic Breeding, Ministry of Agriculture and Fujian Agriculture and Forestry University, Fuzhou 350002, China

## Abstract

Population structure determines sugarcane yield, of which canopy structure is a key component. To fully understand the relations between sugarcane yield and parameters of the canopy structure, 17 sugarcane varieties were investigated at five growth stages. The results indicated that there were significant differences between characterized parameters among sugarcane populations at different growth stages. During sugarcane growth after planting, leaf area index (LAI) and leaf distribution (LD) increased, while transmission coefficient for diffuse radiation (TD), mean foliage inclination angle (MFIA), transmission coefficient for solar beam radiation penetration (TR), and extinction coefficient (*K*) decreased. Significant negative correlations were found between sugarcane yield and MFIA, TD, TR, and *K* at the early elongation stage, while a significant positive correlation between sugarcane yield and LD was found at the same stage. A regression for sugarcane yield, with relative error of yield fitting less than 10%, was successfully established: sugarcane yield = 2380.12 + 46.25 × LD − 491.82 × LAI + 1.36 × MFIA + 614.91 × TD − 1908.05 × TR − 182.53 ×  *K* + 1281.75 × LD − 1.35 × MFIA + 831.2 × TR − 407.8 ×  *K* + 8.21 × MFIA − 834.50 × TD − 1695.49 ×  *K*  (*R* ^2^ = 0.94**).

## 1. Introduction

Sugarcane (*Saccharum* spp. hybrids), with economical importance, has by far the highest yield in crops. Since vegetative stalks are targeted in sugarcane harvest, population structure forms the basis of sugarcane yield. The population structure refers to the dynamics of the distribution and arrangement of each single plant, total leaf area, total plant number, and total root weight in time and space. The crop canopy structure plays a key role in the population structure because it directly affects not only the interception of sunlight but also the photosynthetic efficiency and crop yield of the population through the influence on microenvironment of water, heat, and atmosphere on the canopy [1–3]. Therefore, the light use efficiency of a crop population is directly related to its canopy structure. The biological yield of a crop and its organ distributions are the ultimate results of the canopy structure, which will be reflected in photosynthetic characteristics. There is vital significance for the exploration of appropriate population structure and the cultivation of high-yield and high-quality crops through the study on canopy structure [4]. The leaf morphologies and population structures of other crops such as rice, wheat, and cotton have already been investigated [5–11].

The cultivation of an optimized population structure to improve optical radiation distribution within the canopy and to increase energy utilization is the basis for high yield in crops. The main indices for light radiation within the crop canopy are leaf area index (LAI), mean foliage inclination angle (MFIA), transmission coefficient for diffuse radiation (TD), transmission coefficient for solar beam radiation penetration (TR) in different altitude angles and azimuth angles, extinction coefficient (*K*), and leaf distribution (LD). It is reported there is a close relationship among these indices [5–11]. Canopy light interception can be influenced by the LAI, and it rises along with the increasing LAI. Light interception peaks when LAI is at its optimum. The photosynthetic rate can be affected by the light interception and an appropriate increase in light interception rate of a population can improve the photosynthetic capacity and thus increase production [6–10]. Canopy *K* value at all levels reflects vertical distribution of the leaf area and leaf angle and also the vertical diminishing status of canopy light. In recent years, the investigation on the canopy structure of crops has been greatly facilitated due to the emergence of canopy image analysis techniques. Moreover, the population structure characteristic values such as LAI, MFIA, TD, TR in different altitude angles and azimuth angles, *K*, and LD were successfully determined [9].

Sugarcane is not only a main sugar crop but also an important energy one. The selection of yield and quality-related traits is a key for breeding superior sugarcane varieties. The relationships between leaf morphologies and yields or sucrose contents in sugarcane have been explored [12–15]. However, ecophysiological studies on relationships between characteristics of the canopy structure and sugarcane growth as well as yields have been rarely reported [12–14]. Currently, there is no report on the effects of sugarcane leaf morphology and its spatial distribution on radiation characteristics of canopy.

From all the above, the prediction of sugarcane yield-related traits in population structure at different growth stages is significant in theory and practice for breeding high-yield sugarcane varieties. The canopy spaces and light distributions of different sugarcane populations were investigated in the present study. The relationships between structural characteristics and their yield-related traits were also revealed among different varieties. This study aims to provide references for breeding of high-yield sugarcane varieties on the basis of high-quality population structure.

## 2. Materials and Methods

### 2.1. Materials

The tested sugarcane varieties were FN94-0403, FN94-0744, FN95-1726, FN96-0907, FN98-10100, ROC10, GT94-116, GT95-118, GT96-211, GT96-44, GT97-18, MT70-611, YT92-1287, YT96-107, YT9-6794, YT96-835, and YT96-86. These varieties were planted in the farm of Sugarcane Research Institute, Fujian Agriculture and Forestry University (longitude: 119.23 E, latitude: 26.08 N), as a randomized complete block design. There were three rows for each variety, with the amount of 45,000 sugarcane two-bud sets per hm^−2^. The plot area was 31.35 m^2^, with the row length of 9.5 meter and the row space of 1.1 meter. Field management level was slightly higher than that of the local production fields, with timely cultivator earth, fertilization, irrigation, and pest control. During field management, the same pilot and the same technical measure should be completed on the same day.

### 2.2. Methods

The canopy structure parameters of sugarcane were determined by CI-100 digital plant canopy imager (Washington, USA) at the stages of seedling (May), tillering (June), early elongation (July), rapid elongation (August), and late elongation (September), respectively. The measured indices include LAI, MFIA, TD, TR, *K*, and LD. Fifteen plants of each variety were randomly selected for measurement on canopy structure parameters with three replicates. In mid-November, plant height, stalk diameter, and stalk number for all tested varieties were measured. Single stem weight and cane yield were calculated according to the following formulas: single stem weight = 0.785 × plant height × stalk diameter^2^, cane yield = single stem weight × effective stalk number per unit area.

The measurement was conducted according to established methods [1–3]. During evening time when sunshine was not particularly strong and the sky remained cloudless, observation stick with a fish-eye probe was installed centrally between the rows and was adjusted to exclude any influence from the outside including shadows. Five images were taken in each district. Software for plant canopy analysis, provided by U.S. CID, Inc. (Washington, USA), was applied to calculate the canopy structure parameters. During measurement, LD was represented by distribution frequency of the leaf within each azimuth. The leaf blade azimuth is the angle between the normal direction of leaf surface projection and the direction North; this was then divided clockwise into four directions 90° between each other. *K* and TR were derived according to zenith angle, which was the angle between the target direction and the zenith direction. In this study, CI100 fish-eye lens were set as the observation point, and four angles, 27°, 45°, 63°, and 81°, were determined from the beginning of 9° to 18° zenith angle.

### 2.3. Statistical Analyses

The variance analysis, factor analysis, and regression analysis were performed on the means of data by DPS statistical software [16].

## 3. Result and Analysis

### 3.1. Seasonal Changes of Population Structure Characteristics of Sugarcane Varieties

#### 3.1.1. Leaf Area Index (LAI)

Sugarcane LAIs varied significantly at different growth stages. As seasons change, the LAIs of all varieties increased (Table 1). There was no significant difference in LAI between sugarcane varieties at seedling (May) and tillering stages (June), but at the early elongation stage (July) there was a significant difference. The average LAIs of FN94-0403, FN98-10100, GT96-44, GT94-116, FN96-0907 and MT70-611 at the early elongation stage (July) were larger than those of YT96-794, YT96-835, YT96-107, and GT95-118. There was a relatively small difference in LAI between sugarcane varieties at the rapid elongation stage (August) and the late elongation stage (September). At the rapid elongation stage, the largest average LAI was MT70-611, while the smallest average LAIs were YT96-794 and ROC10. At the late elongation stage (September), the largest average LAIs were GT95-118 and FN98-10100, while the smallest average LAI was YT96-794.

#### 3.1.2. Mean Foliage Inclination Angle (MFIA)

There were significant differences in the sugarcane MFIAs at different growth stages (Table 2). As seasons changed, the maximum MFIA appeared at the seedling stage (May), while the minimum one appeared at the rapid elongation stage (August). There was no significant difference in MFIA between sugarcane varieties at seedling stage (May), tillering stage (June), and the rapid elongation stage (August), but there was a significant difference at the early elongation stage (July). Compared to those of GT95-118, ROC10, and FN95-1726 at the early elongation stage (July), the MEIAs of FN94-0403 and MT70-611 were smaller. There were large differences in the average MFIA among sugarcane varieties at the late elongation stage (September). The MFIAs of three sugarcane varieties, FN96-0907, GT97-18, and FN94-0403, were larger than those of another two sugarcane varieties, GT96-44 and YT96-107.

#### 3.1.3. Transmission Coefficient for Diffuse Radiation (TD)

There were significant differences in the sugarcane TD at different growth stages (Table 3). As seasons changed, the maximum TD appeared at the seedling stage (May), while the minimum TD was found at the late elongation stage (September). There was no significant difference in TD between sugarcane varieties at the seedling stage, the tillering stage (June), the rapid elongation stage (August), and the late elongation stage (September), but there was a significant difference at the early elongation stage (July). The TD of GT95-118 at the early elongation stage (July) was larger than other sugarcane varieties including FN98-10100.

#### 3.1.4. Transmission Coefficient for Solar Beam Radiation Penetration (TR)

The incident light, reflecting solar radiation, can be considered as parallel light beam reaching ground, which is the major light component of plant photosynthesis. The distribution of incident light in a population is determined by the population structure. It is generally considered that light decreases from top to bottom layers of the population, and even the lowest layer of the population would receive this light. The TR reflects the distribution of incident light in the crop population, relating to the transmittance of crop population and the interception of light. Effects were extremely significant on TR from different sugarcane varieties, growth stages, zenith angles, variety × zenith angle, and growth stage × zenith angle. TR decreased linearly with increasing the zenith angle from top to bottom in the vertical direction (Table 4). When the zenith angles were 9° and 27°, respectively, there was little difference in TR. However, when the zenith angle reached 63°, TR decreased sharply. When the zenith angle increased from 9° to 81°, TR decreased from 0.23 to 0.04. The results indicated that the distribution and transmission of light in sugarcane population decreased gradually from top to bottom, while the interception of light increases from top to bottom. There were significant differences in TRs of sugarcane varieties at different growth stages. With a change of seasons, the maximum TR appeared at the seedling stage (May), while the minimum TR appeared at the late elongation stage (September). There were significant differences in the TR between sugarcane varieties at each growth stage. Among all tested sugarcane varieties, GT97-18, FN95-1726, and YT92-1287 had the largest TRs at the seedling stage (May), and GT96-211 had the smallest. Similarly, FN98-10100, MT70-611, and YT96-794 had the largest TRs and YT92-1287 had the smallest at the tillering stage (June). GT95-118 and ROC10 had the largest TRs, while FN94-0403 and FN98-10100 had the smallest at the early elongation stage (July). ROC10 and YT96-794 had the largest TRs, while FN94-0744 and MT70-611 had the smallest at the rapid elongation stage (August). FN94-0403 and YT96-794 had larger TRs than those of FN98-10100 and GT95-118 at the late elongation stage (September).

#### 3.1.5. Extinction Coefficient (*K*)

The extinction coefficient (*K*) represents the attenuation characteristic parameters of the light intensity in the crop population in the vertical direction [2]. *K*_1_ to *K*_5_ represent different *K* values of canopy at different altitude angles (0~90°). As indicated in Table 5, *K* increased with an increasing altitude angle from top to bottom in the vertical direction. When the altitude angles were 9° and 27°, respectively, the difference of *K* was small. When the altitude angle reached 45°, *K* increased sharply. It should be noted that there were significant differences in *K* values at different growth stages. As seasons changed, the maximum *K* appeared at the seedling stage (May) and the minimum at the late elongation stage (September). However, there was no significant difference in the *K* between sugarcane varieties at different growth stages.

#### 3.1.6. Leaf Distribution (LD)

Leaf distribution (LD) refers to the scattering density in leaves at each direction. This index characterizes the spatial distribution characteristics of the canopy leaves of the crop population. There were significant differences in LD at different azimuth angles (Table 6). At the seedling stage (May), the tillering stage (June), the early elongation stage (July), and the rapid elongation stage (August), the LDs at 270~360° azimuth angle were the highest, followed by those at 180~270°, while those at 90~180° were the lowest. However, at the late elongation stage (September), the LDs at 0~90° azimuth angle were the highest, while those at 180~270° were the lowest. There were significant differences in LDs at different growth stages. As seasons changed, the highest LDs appeared at the seedling stage (May) and the lowest at the late elongation stage (September). Within some stages like the seedling (May) and the early elongation (July), there were significant differences between some sugarcane varieties. For example, FN98-10100 and GT96-211 had significantly higher LDs than that of GT97-18 at the seedling stage (May), and FN98-10100 had a higher LD than that of GT95-118 at the early elongation stage (July). There was no significant difference in LD between sugarcane varieties at the tillering stage (June), the rapid elongation stage (August), and the late elongation stage (September).

### 3.2. Factor and Regression Analyses of Canopy Structure Parameters and Their Correlation with Yield-Related Traits in Sugarcane

From thirty canopy parameters of sugarcane at five stages, the first six principal factors related to four yield-related traits were characterized, the cumulative contribution rates were generated, and the initial factor loading matrix is presented in Table 7. The cumulative contribution rate of the characteristic values of the first six principal factors reached 90%. The main information contained in the first six principal factors represent most of the information from the thirty canopy parameters and the initial characteristic parameters of the four yield-related traits. The selection of these 6 factors not only preserved the major part of the information but also achieved the goal of dimensionality reduction. The communalities of the traits were listed in Tables 8 and 9. The communality of the *f*th trait is the contribution of the total principal factors to the total variance of the *i*th trait. The larger the communality is, the better the representativeness of this principal factor to the variable is.

The communalities of 34 traits were large, indicating that the six principal factors were well representative of these 34 traits after performing the varimax rotation, which maximized the total variance of each factor in the loading matrix. The factor loading matrix by varimax rotation of the 6 principal factors was shown in Table 8. The factor loading matrix after varimax rotation had significantly increased loading values of the important variables represented by the principal factors (refer to the initial loading values in Table 7). The principal factors become more biologically meaningful after varimax rotation. As shown in Table 8, the traits with the major roles in the first principal factor were the MFIA and *K* at the early elongation stage; LAI, MFIA, TD, TR, and LD at the rapid elongation stage; and TR at the late elongation stage. Their loading values were large. The loading values of LAI, M FIA, TD, TR, *K*, and LD at the seedling stage were larger than others in the second principal factor. The loading values of LAI, M FIA, TD, TR, *K*, and LD and stalk number per hectare at the early elongation stage were larger than others in the third principal factor. The loading values of LAI, MFIA, TD, TR, *K*, and LD at the tillering stage were larger than others in the fourth principal factor. The loading values of *K* at the rapid elongation stage, LAI and TD at the late elongation stage, and the plant height were larger in the fifth principal factor. The loading values of MFIA and *K* at the late elongation stage, stem diameter, single-stalk weight, and sugarcane yield were larger in the sixth principal factor. There were significant negative correlations between the plant height and the MFIA, between the plant height and the *K* at the rapid elongation stage, and between the plant height and the TR at the late elongation stage, while there were significant positive correlations between the MFIA and TD at the late elongation stage. There were significant negative correlations between the stalk number per hectare and TD or TR at the early elongation stage, while there were significant positive correlations between stalk number per hectare and the LAI or LD at the early elongation stage. There was a significant negative correlation between the stem diameter and LD at tillering stage. There were no significant correlations between stem diameter and the MFIA or *K* at the late elongation stage. There was a significant negative correlation between the single-stalk weight and LD at tillering stage, while there were significant positive correlations between single-stalk weight and the MFIA or *K* at the late elongation stage. There were significant negative correlations between the sugarcane yield and the MFIA, TD, TR, or *K* at early elongation stage, while there was a significant positive correlation of the sugarcane yield with the LD of the early elongation stage. There were correlations of sugarcane yield with the MFIA and *K* at the late elongation stage, but they were not significant. A regression analysis was also conducted for the canopy structure parameters and their correlation with yield-related traits in sugarcane. The sugarcane yield was predicted by the canopy structure characteristic parameters. The regression equation was as follows: sugarcane yield = 2380.12 + 46.25 × LD (tillering stage) − 491.82 × LAI (early elongation stage) + 1.36 × MFIA (early elongation stage) + 614.91 × TD (early elongation stage) − 1908.05 × TR (early elongation stage) − 182.53 ×  *K* (early elongation stage) + 1281.75 × LD (early elongation stage) − 1.35 × MFIA (rapid elongation stage) + 831.2 × TR (rapid elongation stage) − 407.8 ×  *K* (rapid elongation stage) + 8.21 × MFIA (late elongation stage) − 834.50 × TD (late elongation stage) − 1695.49 ×  *K* (late elongation stage) (*R* ^2^ = 0.94**). The relative error of yield fitting for all these 17 test sugarcane varieties was less than 10%.

## 4. Discussion and Conclusions

Crop yield mainly depends on the light receiving ability and light distribution in the crop population. The leaves are the main organ for photosynthesis. Therefore, the characteristics of leaves are important in light distribution and photosynthetic traits of a crop's population [1–4]. The shape, size, quantity, and spatial distribution of leaves determine light use efficiency and influence light environment and vice versa. Sugarcane varieties with short, narrow, thick, and straight canopy have high-yield potentials [12]. Leaf morphology and canopy structure directly affect light interception of canopy and light distribution at different layers [6–10]. Leaf morphology and spatial distribution influence radiation characteristics of the canopy [17–19]. In the present study, large radiation parameters in canopy, especially in lower layers of the canopy, were found in sugarcane genotypes with short, narrow, and straight leaves, which is beneficial to light absorption, resulting in favorable conditions for sugarcane yield and sugar accumulation during the later growth period.

Appropriate population structure is essential to improve energy use efficiency and thus the high crop yield. A positive correlation was found between proper LAI and dry matter accumulation [20]. Enhancement of population LAI, especially at the later development stages, can increase dry matter accumulation. MFIA influences the plant ability to receive solar radiation and the canopy distribution, especially on *K* values. By breeding varieties with proper leaf angles and distribution, an ideal population structure can be obtained with the most effective leaf area. Zhang et al. [21] revealed that the canopy MFIA of winter wheat decreases with crop growth along with increasing nitrogen application. Even with the same LAI on two populations, different light distributions, canopy photosynthetic rates, and dry matter accumulations can be observed if spatial distribution of leaves and average leaf angles are different [20]. Numeral researches have proved that crop productions tightly correlate with the functions of canopy populations [20–22]. Maddonni et al. [22] pointed out that light attenuation of maize, a row crop, was influenced by canopy architecture in terms of size, shape, and orientation of shoot components. Monsi and Saeki [23] showed that, under full light conditions, canopy photosynthesis was maximized at a high LAI with vertically inclined leaves, while, under low light conditions, it was at a low LAI with horizontal leaves. They suggested that plants have evolved standing stem with different orientations of branches and leaves to maximize canopy photosynthesis. Canopy photosynthesis models are useful to analyze the size structure of populations in plant communities and to predict the structure and function of future terrestrial ecosystems [24]. Stewart et al. [25] found that plants with very upright leaves can have both the smallest and largest daily canopy photosynthesis, depending on row widths. Reta-Sánchez and Fowler [26] revealed that early canopy modifications simulating plant characteristics such as reduced plant height, short branches, and modified leaf shape increased light availability at the medium and upper part of the canopy. Liu et al. [27] demonstrated that narrow-row planting patterns improved the canopy structure, allowed more IPAR (light intercepted by a canopy) to reach the middle-row strata of the canopy, and enhanced the leaf photosynthetic characteristics of maize crops at silking stage compared with the control. As illustrated in the present study, sugarcane canopy structure can be affected by planting pattern and other physiological characters such as light interception and radiation use efficiency. Besides, a large crop photosynthetic area at the late growth stage is one of the most important features of population structure on productive sugarcane farmland, which are similar to the previous reports [20–27].

Canopy structure, reflected by vertical and spatial distribution, orientation, and density of foliage and its supporting structures, determines the pattern of light attenuation, the distribution of photosynthesis, respiration, transpiration, and nutrient cycling in the canopy and therefore the yield and quantity of crops [28]. The present study indicated that different influences of canopy structure on sugarcane yield component factors at different stages. The mutual influences on each factor were reflected by the correlations of the parameters in the canopy structure. Understanding these influences helps the breeders to select varieties in optimization of the canopy structure at each developmental stage and thus helps the sugarcane farmers in their cultivation and yield prediction. The correlation and regression models established in this study demonstrated that the canopy structure at the early elongation stage is closely related with the formation of sugarcane yield because the sugarcane varieties with small MFIA, TD, TR, *K*, and large LD at the early elongation stage have the greatest number of leaves in unit space and the spatial distributions of leaves are uniform. Ecologically, this kind of parameter combination is in favor of the interception of light by the sugarcane population and the improvement of light energy utilization. Physiologically, more photosynthetic products could be accumulated in this condition, which would generate high-yield sugarcane population based on a solid foundation of a high-quality canopy structure for sugarcane growth and development [17, 28]. What should also be stressed is that all the 17 sugarcane varieties are popular currently in China and they do cover a relatively wide range in population structure for making regression, which should help to distinguish between different types of sugarcane varieties and canopy structure characteristics and thereby reduce the test result errors and ensure the reliability of the regression for sugarcane yield established in this study.

Given that the organic substances required for sugarcane growth are produced by sugarcane leaves through photosynthesis, the canopy structure and light distribution of sugarcane at the elongation stage thus play a crucial role in the structure formation, the accumulation and partitioning of photosynthetic products, and thus the yield formation. Our study clearly demonstrates this phenomenon by the correlations between the main canopy structure parameters at the early elongation stage and sugarcane yield. As seasons changed, the LAI and LD increase, while the TD, MFIA, TR, and *K* decrease, which is in accordance with previous reports [17–19, 28]. Attention to some traits, which are negatively correlated with yields at specific growth stages, is required in breeding and cultivation. When selecting the yield-related traits based on the canopy structure parameters of sugarcane, the values for the above mentioned traits are still required to be optimized. For example, the mutual shading between leaves should be avoided. In short, it is significant in breeding and cultivation of high-yield and high-quality sugarcane varieties through the rapid and accurate confirmation of characteristics of canopy structure and timely optimization of the population size.

## Figures and Tables

**Table 1:** Seasonal changes of leaf area index in sugarcane varieties.

Varieties	Seedling	Tillering	Early elongation	Rapid elongation	Late elongation	Mean
FN94-0403	0.640 ± 0.036^a^	0.990 ± 0.181^a^	1.487 ± 0.230^a^	1.550 ± 0.250^ab^	1.827 ± 0.097^bc^	1.299 ± 0.466^ab^
FN94-0744	0.587 ± 0.068^a^	0.880 ± 0.148^a^	1.160 ± 0.214^bcde^	1.597 ± 0.285^ab^	1.913 ± 0.117^abc^	1.227 ± 0.518^ab^
FN95-1726	0.603 ± 0.125^a^	0.877 ± 0.050^a^	1.050 ± 0.072^de^	1.677 ± 0.222^ab^	2.113 ± 0.183^abc^	1.264 ± 0.585^ab^
FN96-0907	0.783 ± 0.363^a^	0.870 ± 0.170^a^	1.247 ± 0.064^abcde^	1.443 ± 0.231^ab^	1.983 ± 0.257^abc^	1.265 ± 0.491^ab^
FN98-10100	0.730 ± 0.105^a^	0.873 ± 0.186^a^	1.420 ± 0.035^ab^	1.560 ± 0.178^ab^	2.327 ± 0.064^ab^	1.382 ± 0.597^a^
GT94-116	0.720 ± 0.187^a^	1.050 ± 0.254^a^	1.323 ± 0.086^abcd^	1.703 ± 0.562^ab^	2.037 ± 0.138^abc^	1.367 ± 0.543^a^
GT95-118	0.777 ± 0.296^a^	0.937 ± 0.045^a^	0.987 ± 0.316^e^	1.420 ± 0.295^ab^	2.417 ± 0.568^a^	1.307 ± 0.681^ab^
GT96-211	0.833 ± 0.296^a^	0.940 ± 0.070^a^	1.180 ± 0.125^bcde^	1.497 ± 0.237^ab^	2.013 ± 0.294^abc^	1.293 ± 0.480^ab^
GT96-44	0.637 ± 0.060^a^	0.997 ± 0.155^a^	1.367 ± 0.076^abc^	1.630 ± 0.040^ab^	2.047 ± 0.242^abc^	1.335 ± 0.519^a^
GT97-18	0.593 ± 0.214^a^	1.030 ± 0.165^a^	1.127 ± 0.211^cde^	1.430 ± 0.061^ab^	2.007 ± 0.195^abc^	1.237 ± 0.508^ab^
MT70-611	0.623 ± 0.121^a^	0.843 ± 0.154^a^	1.267 ± 0.096^abcde^	1.887 ± 0.287^a^	1.893 ± 0.408^abc^	1.303 ± 0.578^ab^
ROC10	0.617 ± 0.180^a^	0.963 ± 0.199^a^	1.063 ± 0.087^de^	1.280 ± 0.095^b^	2.090 ± 0.200^abc^	1.203 ± 0.528^ab^
YT92-1287	0.550 ± 0.078^a^	1.180 ± 0.403^a^	1.153 ± 0.154^bcde^	1.450 ± 0.218^ab^	1.993 ± 0.112^abc^	1.265 ± 0.520^ab^
YT96-107	0.617 ± 0.075^a^	1.020 ± 0.149^a^	1.027 ± 0.085^e^	1.543 ± 0.288^ab^	2.103 ± 0.266^abc^	1.262 ± 0.556^ab^
YT96-794	0.743 ± 0.144^a^	0.827 ± 0.112^a^	1.023 ± 0.106^e^	1.323 ± 0.154^b^	1.777 ± 0.392^c^	1.139 ± 0.428^b^
YT96-835	0.603 ± 0.064^a^	0.973 ± 0.352^a^	0.993 ± 0.090^e^	1.447 ± 0.240^ab^	2.063 ± 0.289^abc^	1.216 ± 0.556^ab^
YT96-86	0.667 ± 0.100^a^	1.023 ± 0.278^a^	1.087 ± 0.080^de^	1.457 ± 0.145^ab^	2.117 ± 0.266^abc^	1.270 ± 0.535^ab^

Mean	0.666 ± 0.165^e^	0.957 ± 0.190^d^	1.174 ± 0.193^c^	1.523 ± 0.2521^b^	2.042 ± 0.272^a^	1.273

Notes: the lowercase letters denote significant differences at the level of 0.05.

**Table 2:** Seasonal changes of mean foliage inclination angle in sugarcane varieties.

Varieties	Seedling	Tillering	Early elongation	Rapid elongation	Late elongation	Mean
FN94-0403	75.09 ± 6.97^a^	52.39 ± 12.42^ab^	43.27 ± 13.59^c^	28.35 ± 7.47^a^	47.33 ± 5.41^a^	49.29 ± 17.74^a^
FN94-0744	82.39 ± 4.36^a^	53.76 ± 12.23^ab^	50.56 ± 11.90^bc^	25.69 ± 5.43^a^	45.57 ± 14.80^ab^	51.59 ± 20.88^a^
FN95-1726	74.18 ± 10.19^a^	53.77 ± 2.60^ab^	62.94 ± 6.55^ab^	38.58 ± 19.84^a^	42.72 ± 3.27^abc^	54.44 ± 16.19^a^
FN96-0907	68.29 ± 28.34^a^	52.81 ± 7.15^ab^	55.99 ± 12.51^abc^	31.16 ± 8.58^a^	49.40 ± 1.97^a^	51.53 ± 17.61^a^
FN98-10100	68.57 ± 15.71^a^	64.93 ± 11.70^a^	50.19 ± 9.93^bc^	28.77 ± 5.09^a^	40.31 ± 1.62^abc^	50.55 ± 17.64^a^
GT94-116	67.91 ± 21.94^a^	53.79 ± 14.77^ab^	49.82 ± 4.23^bc^	25.39 ± 16.63^a^	34.08 ± 14.58^abc^	46.20 ± 20.32^a^
GT95-118	58.86 ± 27.72^a^	50.71 ± 11.38^ab^	71.29 ± 11.89^a^	32.53 ± 10.94^a^	38.29 ± 1.29^abc^	50.34 ± 19.35^a^
GT96-211	55.65 ± 16.30^a^	48.02 ± 12.63^ab^	60.37 ± 4.78^abc^	27.66 ± 4.59^a^	38.05 ± 6.35^abc^	45.95 ± 14.95^a^
GT96-44	69.06 ± 6.96^a^	46.22 ± 10.18^ab^	57.23 ± 4.90^abc^	28.17 ± 1.42^a^	30.41 ± 2.62^bc^	46.22 ± 16.96^a^
GT97-18	75.23 ± 12.73^a^	43.45 ± 3.51^ab^	58.64 ± 13.19^abc^	34.58 ± 0.41^a^	47.57 ± 8.59^a^	51.89 ± 16.44^a^
MT70-611	76.13 ± 16.93^a^	56.13 ± 4.22^ab^	43.16 ± 5.30^c^	20.75 ± 2.78^a^	41.91 ± 13.42^abc^	47.62 ± 20.74^a^
ROC10	71.05 ± 25.73^a^	46.34 ± 9.53^ab^	67.57 ± 1.03^ab^	39.81 ± 12.27^a^	36.13 ± 9.34^abc^	52.18 ± 19.08^a^
YT92-1287	75.74 ± 9.93^a^	39.42 ± 15.36^b^	52.96 ± 11.11^abc^	29.31 ± 5.14^a^	41.12 ± 3.70^abc^	47.71 ± 18.49^a^
YT96-107	79.04 ± 6.35^a^	45.82 ± 11.75^ab^	61.00 ± 16.68^abc^	30.50 ± 10.87^a^	27.13 ± 11.93^c^	48.70 ± 22.46^a^
YT96-794	59.10 ± 26.22^a^	52.69 ± 8.61^ab^	60.15 ± 12.02^abc^	36.99 ± 5.01^a^	39.21 ± 4.37^abc^	49.63 ± 15.43^a^
YT96-835	75.54 ± 8.98^a^	51.18 ± 26.61^ab^	58.57 ± 7.32^abc^	36.83 ± 13.14^a^	34.42 ± 2.88^abc^	51.31 ± 19.73^a^
YT96-86	72.13 ± 5.20^a^	47.15 ± 14.81^ab^	54.40 ± 5.16^abc^	34.56 ± 9.72^a^	36.71 ± 9.20^abc^	48.99 ± 16.22^a^

Mean	70.82 ± 15.65^a^	50.50 ± 11.67^c^	56.36 ± 11.06^b^	31.15 ± 9.47^e^	39.43 ± 9.01^d^	

Notes: the lowercase letters denote significant differences at the level of 0.05.

**Table 3:** Seasonal changes of transmission coefficient for diffuse radiation in sugarcane varieties.

Varieties	Seedling	Tillering	Early elongation	Rapid elongation	Late elongation	Mean
FN94-0403	0.63 ± 0.03^a^	0.45 ± 0.08^a^	0.33 ± 0.06^ab^	0.27 ± 0.06^a^	0.23 ± 0.01^a^	0.38 ± 0.16^ab^
FN94-0744	0.69 ± 0.07^a^	0.49 ± 0.05^a^	0.39 ± 0.07^ab^	0.27 ± 0.08^a^	0.23 ± 0.03^a^	0.42 ± 0.18^ab^
FN95-1726	0.69 ± 0.12^a^	0.50 ± 0.06^a^	0.44 ± 0.02^ab^	0.29 ± 0.04^a^	0.21 ± 0.04^a^	0.42 ± 0.18^ab^
FN96-0907	0.64 ± 0.16^a^	0.48 ± 0.07^a^	0.37 ± 0.02^ab^	0.29 ± 0.06^a^	0.24 ± 0.05^a^	0.40 ± 0.17^ab^
FN98-10100	0.60 ± 0.04^a^	0.51 ± 0.11^a^	0.32 ± 0.04^b^	0.26 ± 0.01^a^	0.17 ± 0.02^a^	0.37 ± 0.17^b^
GT94-116	0.63 ± 0.07^a^	0.44 ± 0.07^a^	0.37 ± 0.04^ab^	0.27 ± 0.11^a^	0.21 ± 0.04^a^	0.38 ± 0.16^ab^
GT95-118	0.60 ± 0.15^a^	0.46 ± 0.03^a^	0.51 ± 0.13^a^	0.32 ± 0.04^a^	0.16 ± 0.06^a^	0.41 ± 0.18^ab^
GT96-211	0.57 ± 0.12^a^	0.48 ± 0.01^a^	0.41 ± 0.07^ab^	0.27 ± 0.05^a^	0.20 ± 0.03^a^	0.39 ± 0.15^ab^
GT96-44	0.65 ± 0.02^a^	0.44 ± 0.07^a^	0.37 ± 0.04^ab^	0.26 ± 0.03^a^	0.21 ± 0.03^a^	0.38 ± 0.16^ab^
GT97-18	0.70 ± 0.11^a^	0.43 ± 0.04^a^	0.44 ± 0.11^ab^	0.32 ± 0.02^a^	0.22 ± 0.04^a^	0.42 ± 0.18^ab^
MT70-611	0.64 ± 0.09^a^	0.51 ± 0.06^a^	0.37 ± 0.02^ab^	0.22 ± 0.04^a^	0.23 ± 0.07^a^	0.40 ± 0.18^ab^
ROC10	0.68 ± 0.10^a^	0.46 ± 0.05^a^	0.47 ± 0.03^ab^	0.32 ± 0.04^a^	0.20 ± 0.04^a^	0.43 ± 0.17^a^
YT92-1287	0.70 ± 0.05^a^	0.42 ± 0.07^a^	0.41 ± 0.05^ab^	0.29 ± 0.06^a^	0.20 ± 0.01^a^	0.40 ± 0.18^ab^
YT96-107	0.66 ± 0.02^a^	0.41 ± 0.05^a^	0.45 ± 0.10^ab^	0.27 ± 0.07^a^	0.17 ± 0.04^a^	0.39 ± 0.18^ab^
YT96-794	0.61 ± 0.12^a^	0.48 ± 0.06^a^	0.44 ± 0.06^ab^	0.31 ± 0.03^a^	0.23 ± 0.05^a^	0.42 ± 0.15^ab^
YT96-835	0.66 ± 0.04^a^	0.47 ± 0.16^a^	0.45 ± 0.06^ab^	0.30 ± 0.04^a^	0.19 ± 0.02^a^	0.41 ± 0.18^ab^
YT96-86	0.63 ± 0.03^a^	0.47 ± 0.04^a^	0.43 ± 0.04^ab^	0.28 ± 0.03^a^	0.18 ± 0.03^a^	0.40 ± 0.17^ab^

Mean	0.65 ± 0.08^a^	0.46 ± 0.06^b^	0.41 ± 0.07^c^	0.28 ± 0.05^d^	0.20 ± 0.04^e^	

Notes: the lowercase letters denote significant differences at the level of 0.05.

**Table 4:** Seasonal changes of transmission coefficient for solar beam radiation penetration in sugarcane varieties.

Varieties	Seedling	Tillering	Early elongation	Rapid elongation	Late elongation	Mean
FN94-0403	0.574 ± 0.28^abcd^	0.415 ± 0.21^abcde^	0.277 ± 0.16^f^	0.228 ± 0.11^bcd^	0.216 ± 0.13^a^	0.342 ± 0.23^cd^
FN94-0744	0.616 ± 0.32^ab^	0.445 ± 0.21^abcd^	0.354 ± 0.19^cde^	0.217 ± 0.10^cd^	0.201 ± 0.13^abc^	0.367 ± 0.25^abcd^
FN95-1726	0.627 ± 0.30^a^	0.448 ± 0.20^abcd^	0.410 ± 0.23^abc^	0.268 ± 0.15^abc^	0.169 ± 0.10^abcd^	0.384 ± 0.26^a^
FN96-0907	0.551 ± 0.31^bcd^	0.443 ± 0.21^abcd^	0.351 ± 0.20^cde^	0.257 ± 0.13^abc^	0.193 ± 0.12^abcd^	0.359 ± 0.24^abcd^
FN98-10100	0.545 ± 0.27^bcd^	0.469 ± 0.25^a^	0.291 ± 0.16^ef^	0.228 ± 0.10^bcd^	0.141 ± 0.09^cd^	0.335 ± 0.24^d^
GT94-116	0.567 ± 0.29^abcd^	0.409 ± 0.21^abcde^	0.325 ± 0.17^*def*^	0.223 ± 0.15^bcd^	0.169 ± 0.10^abcd^	0.339 ± 0.24^d^
GT95-118	0.535 ± 0.29^cd^	0.418 ± 0.20^abcde^	0.460 ± 0.28^a^	0.275 ± 0.13^abc^	0.127 ± 0.08^d^	0.363 ± 0.25^abcd^
GT96-211	0.511 ± 0.25^d^	0.416 ± 0.19^abcde^	0.374 ± 0.21^bcd^	0.238 ± 0.10^abcd^	0.167 ± 0.09^abcd^	0.341 ± 0.22^cd^
GT96-44	0.587 ± 0.27^abc^	0.393 ± 0.19^cde^	0.322 ± 0.19^*def*^	0.225 ± 0.09^bcd^	0.153 ± 0.08^abcd^	0.336 ± 0.23^d^
GT97-18	0.635 ± 0.31^a^	0.388 ± 0.17^cde^	0.381 ± 0.23^bcd^	0.270 ± 0.12^abc^	0.193 ± 0.13^abcd^	0.373 ± 0.25^abc^
MT70-611	0.563 ± 0.30^abcd^	0.466 ± 0.22^ab^	0.316 ± 0.15^*def*^	0.173 ± 0.07^d^	0.205 ± 0.14^abc^	0.345 ± 0.24^bcd^
ROC10	0.603 ± 0.30^abc^	0.419 ± 0.19^abcde^	0.425 ± 0.25^ab^	0.299 ± 0.15^a^	0.158 ± 0.09^abcd^	0.381 ± 0.25^a^
YT92-1287	0.625 ± 0.30^a^	0.349 ± 0.18^e^	0.371 ± 0.20^bcd^	0.247 ± 0.11^abc^	0.173 ± 0.10^abcd^	0.353 ± 0.24^abcd^
YT96-107	0.602 ± 0.30^abc^	0.386 ± 0.18^de^	0.415 ± 0.23^abc^	0.239 ± 0.13^abcd^	0.143 ± 0.07^bcd^	0.357 ± 0.25^abcd^
YT96-794	0.545 ± 0.29^bcd^	0.458 ± 0.21^abc^	0.409 ± 0.23^abc^	0.291 ± 0.13^ab^	0.215 ± 0.12^ab^	0.384 ± 0.23^a^
YT96-835	0.599 ± 0.28^abc^	0.423 ± 0.24^abcd^	0.419 ± 0.22^abc^	0.276 ± 0.14^abc^	0.157 ± 0.09^abcd^	0.375 ± 0.25^ab^
YT96-86	0.577 ± 0.28^abcd^	0.397 ± 0.20^bcde^	0.383 ± 0.20^bcd^	0.265 ± 0.12^abc^	0.155 ± 0.09^abcd^	0.356 ± 0.23^abcd^

Mean	0.580 ± 0.28^a^	0.420 ± 0.20^b^	0.370 ± 0.21^c^	0.248 ± 0.12^d^	0.173 ± 0.10^e^	

TR_1_	0.790^a^	0.587^a^	0.569^a^	0.333^a^	0.264^a^	0.508 ± 0.23^a^
TR_2_	0.796^a^	0.555^a^	0.518^b^	0.321^ab^	0.246^a^	0.487 ± 0.22^b^
TR_3_	0.716^b^	0.497^b^	0.435^c^	0.295^b^	0.207^b^	0.430 ± 0.19^c^
TR_4_	0.486^c^	0.376^c^	0.288^d^	0.235^c^	0.132^c^	0.303 ± 0.13^d^
TR_5_	0.114^d^	0.086^d^	0.038^e^	0.058^d^	0.014^d^	0.062 ± 0.04^e^

Notes: (1) TR_1_, TR_2_, TR_3_, TR_4_, and TR_5_ represent the transmission coefficients for solar beam radiation penetration at the elevation angles of 9°, 27°, 45°, 63°, and 81°, respectively. (2) The lowercase letters denote significant differences at the level of 0.05.

**Table 5:** Seasonal changes of extinction coefficient in sugarcane varieties.

Varieties	Seedling	Tillering	Early elongation	Rapid elongation	Late elongation	Mean
FN94-0403	1.245 ± 1.31	1.161 ± 0.93	1.149 ± 0.77	1.079 ± 0.45	1.170 ± 0.83	1.161 ± 0.88
FN94-0744	1.262 ± 1.43	1.183 ± 0.95	1.183 ± 0.91	1.085 ± 0.42	1.162 ± 0.82	1.175 ± 0.94
FN95-1726	1.244 ± 1.30	1.186 ± 0.94	1.218 ± 1.11	1.065 ± 0.59	1.155 ± 0.74	1.174 ± 0.94
FN96-0907	1.219 ± 1.24	1.193 ± 0.95	1.198 ± 1.01	1.107 ± 0.54	1.181 ± 0.87	1.180 ± 0.92
FN98-10100	1.217 ± 1.19	1.223 ± 1.16	1.183 ± 0.90	1.206 ± 0.59	1.146 ± 0.71	1.195 ± 0.91
GT94-116	1.214 ± 1.20	1.181 ± 0.95	1.171 ± 0.87	1.082 ± 0.48	1.119 ± 0.62	1.154 ± 0.84
GT95-118	1.195 ± 1.09	1.177 ± 0.90	1.243 ± 1.29	1.111 ± 0.57	1.137 ± 0.65	1.173 ± 0.92
GT96-211	1.184 ± 0.98	1.167 ± 0.85	1.209 ± 1.06	1.095 ± 0.45	1.135 ± 0.66	1.158 ± 0.81
GT96-44	1.227 ± 1.20	1.167 ± 0.83	1.205 ± 1.02	1.094 ± 0.46	1.103 ± 0.50	1.159 ± 0.83
GT97-18	1.244 ± 1.31	1.155 ± 0.76	1.203 ± 1.04	1.122 ± 0.59	1.171 ± 0.85	1.179 ± 0.92
MT70-611	1.240 ± 1.32	1.197 ± 0.99	1.153 ± 0.75	1.064 ± 0.32	1.150 ± 0.76	1.161 ± 0.87
ROC10	1.228 ± 1.27	1.159 ± 0.81	1.230 ± 1.19	1.142 ± 0.72	1.127 ± 0.63	1.177 ± 0.93
YT92-1287	1.249 ± 1.33	1.139 ± 0.72	1.188 ± 0.95	1.101 ± 0.49	1.149 ± 0.71	1.165 ± 0.86
YT96-107	1.252 ± 1.36	1.161 ± 0.81	1.210 ± 1.09	1.106 ± 0.54	1.091 ± 0.48	1.164 ± 0.90
YT96-794	1.192 ± 1.08	1.186 ± 0.93	1.210 ± 1.07	1.131 ± 0.63	1.139 ± 0.67	1.172 ± 0.87
YT96-835	1.241 ± 1.30	1.171 ± 0.96	1.199 ± 1.02	1.125 ± 0.65	1.122 ± 0.58	1.172 ± 0.92
YT96-86	1.233 ± 1.25	1.169 ± 0.86	1.189 ± 0.94	1.118 ± 0.59	1.131 ± 0.64	1.168 ± 0.87

Mean	1.229 ± 1.21^a^	1.175 ± 0.88^ab^	1.197 ± 0.98^bcde^	1.108 ± 0.53^cd^	1.141 ± 0.68^d^	

*K*_1_	3.511^a^	2.836^a^	3.063^a^	2.068^a^	2.426^a^	2.781 ± 0.66^a^
*K*_2_	1.172^b^	1.079^b^	1.104^b^	1.007^b^	1.032^b^	1.079 ± 0.07^b^
*K*_3_	0.690^c^	0.756^c^	0.733^c^	0.883^c^	0.809^c^	0.774 ± 0.13^c^
*K*_4_	0.458^d^	0.632^d^	0.579^d^	0.801^cd^	0.732^cd^	0.640 ± 0.16^d^
*K*_5_	0.313^e^	0.572^d^	0.504^d^	0.781^d^	0.704^d^	0.575 ± 0.21^e^

Notes: (1) *K*_1_, *K*_2_, *K*_3_, *K*_4_,  and *K*_5_ represent the canopy extinction coefficients at the elevation angles of 9°, 27°, 45°, 63°, and 81°, respectively. (2) The lowercase letters denote significant differences at the level of 0.05.

**Table 6:** Seasonal changes of leaf distribution in sugarcane varieties.

Varieties	Seedling	Tillering	Early elongation	Rapid elongation	Late elongation	Mean
FN94-0403	0.574 ± 0.14^ab^	0.692 ± 0.11^a^	0.783 ± 0.08^ab^	0.809 ± 0.09^a^	0.859 ± 0.05^a^	0.744 ± 0.14^ab^
FN94-0744	0.533 ± 0.16^ab^	0.661 ± 0.08^a^	0.748 ± 0.09^abc^	0.807 ± 0.12^a^	0.856 ± 0.06^a^	0.721 ± 0.16^ab^
FN95-1726	0.513 ± 0.17^ab^	0.658 ± 0.12^a^	0.722 ± 0.09^bc^	0.787 ± 0.06^a^	0.869 ± 0.05^a^	0.710 ± 0.16^b^
FN96-0907	0.562 ± 0.16^ab^	0.676 ± 0.13^a^	0.770 ± 0.09^ab^	0.804 ± 0.10^a^	0.857 ± 0.06^a^	0.734 ± 0.15^ab^
FN98-10100	0.593 ± 0.15^a^	0.673 ± 0.12^a^	0.799 ± 0.06^a^	0.818 ± 0.07^a^	0.898 ± 0.06^a^	0.756 ± 0.15^a^
GT94-116	0.563 ± 0.14^ab^	0.713 ± 0.08^a^	0.761 ± 0.11^ab^	0.807 ± 0.13^a^	0.864 ± 0.06^a^	0.741 ± 0.15^ab^
GT95-118	0.571 ± 0.16^ab^	0.689 ± 0.10^a^	0.684 ± 0.13^c^	0.783 ± 0.13^a^	0.898 ± 0.06^a^	0.725 ± 0.16^ab^
GT96-211	0.591 ± 0.16^a^	0.664 ± 0.13^a^	0.736 ± 0.07^abc^	0.800 ± 0.10^a^	0.864 ± 0.08^a^	0.731 ± 0.15^ab^
GT96-44	0.545 ± 0.13^ab^	0.694 ± 0.10^a^	0.776 ± 0.05^ab^	0.816 ± 0.09^a^	0.858 ± 0.07^a^	0.738 ± 0.14^ab^
GT97-18	0.506 ± 0.14^b^	0.693 ± 0.14^a^	0.719 ± 0.10^bc^	0.782 ± 0.12^a^	0.867 ± 0.05^a^	0.713 ± 0.16^b^
MT70-611	0.562 ± 0.16^ab^	0.650 ± 0.14^a^	0.756 ± 0.12^abc^	0.833 ± 0.08^a^	0.852 ± 0.09^a^	0.731 ± 0.16^ab^
ROC10	0.521 ± 0.15^ab^	0.674 ± 0.15^a^	0.708 ± 0.06^bc^	0.786 ± 0.13^a^	0.866 ± 0.07^a^	0.711 ± 0.16^b^
YT92-1287	0.512 ± 0.15^ab^	0.705 ± 0.17^a^	0.735 ± 0.12^abc^	0.804 ± 0.14^a^	0.876 ± 0.06^a^	0.726 ± 0.18^ab^
YT96-107	0.556 ± 0.17^ab^	0.720 ± 0.15^a^	0.712 ± 0.10^bc^	0.813 ± 0.08^a^	0.873 ± 0.08^a^	0.735 ± 0.16^ab^
YT96-794	0.561 ± 0.20^ab^	0.659 ± 0.17^a^	0.718 ± 0.10^bc^	0.793 ± 0.12^a^	0.848 ± 0.09^a^	0.716 ± 0.17^b^
YT96-835	0.545 ± 0.14^ab^	0.668 ± 0.15^a^	0.715 ± 0.10^bc^	0.803 ± 0.11^a^	0.872 ± 0.06^a^	0.720 ± 0.16^ab^
YT96-86	0.563 ± 0.14^ab^	0.673 ± 0.09^a^	0.726 ± 0.11^abc^	0.803 ± 0.15^a^	0.886 ± 0.05^a^	0.730 ± 0.16^ab^

Mean	0.551 ± 0.15^e^	0.680 ± 0.12^d^	0.739 ± 0.10^c^	0.803 ± 0.11^b^	0.868 ± 0.07^a^	

LD_1_	0.523^c^	0.680^b^	0.733^b^	0.863^a^	0.909^a^	0.742 ± 0.16^d^
LD_2_	0.385^d^	0.584^c^	0.640^c^	0.761^b^	0.876^ab^	0.649 ± 0.19^c^
LD_3_	0.585^b^	0.701^b^	0.776^a^	0.729^b^	0.820^c^	0.722 ± 0.12^b^
LD_4_	0.712^a^	0.755^a^	0.808^a^	0.858^a^	0.868^b^	0.800 ± 0.09^a^

Notes: (1) LD_1_, LD_2_, LD_3_, and LD_4_ represent the leaf distribution at the azimuth angles of 0~90°, 90~180°, 180~270°, and 270~360°, respectively. (2) The lowercase letters denote significant differences at the level of 0.05.

**Table 7:** Loading matrix of characters initial factor.

Stages	No.	F1	F2	F3	F4	F5	F6	JD	SV
Seeding	LAI	−0.042	0.853	0.074	−0.379	0.238	0.073	0.940	0.060
	MFIA	−0.232	−0.780	−0.060	0.462	−0.215	−0.019	0.926	0.074
	TD	0.212	−0.858	0.085	0.311	−0.138	−0.236	0.960	0.040
	TR	0.235	−0.857	−0.019	0.379	−0.120	−0.172	0.979	0.022
	*K*	−0.171	−0.835	−0.040	0.433	−0.176	0.030	0.948	0.052
	LD	−0.422	0.764	−0.171	−0.174	0.117	0.260	0.902	0.098

Tillering	LAI	0.256	−0.548	−0.648	−0.126	0.329	0.181	0.942	0.058
	MFIA	−0.557	0.599	0.204	0.422	−0.131	−0.173	0.936	0.064
	TD	−0.384	0.495	0.622	0.329	−0.226	0.056	0.942	0.058
	TR	−0.388	0.558	0.562	0.271	−0.280	−0.238	0.986	0.014
	*K*	−0.487	0.650	0.247	0.403	−0.183	−0.125	0.932	0.068
	LD	0.165	−0.288	−0.794	−0.180	0.253	−0.069	0.841	0.159

Early elongation	LAI	−0.820	0.037	−0.244	−0.024	0.366	−0.189	0.904	0.096
	MFIA	0.905	0.270	0.038	−0.062	−0.153	−0.046	0.923	0.077
	TD	0.912	−0.001	0.103	−0.092	−0.301	0.134	0.959	0.041
	TR	0.915	0.064	0.155	−0.033	−0.298	0.124	0.970	0.030
	*K*	0.871	0.283	0.058	−0.024	−0.148	−0.034	0.866	0.134
	LD	−0.864	0.061	−0.132	0.131	0.316	−0.175	0.915	0.085

Rapid elongation	LAI	−0.745	−0.119	−0.144	0.005	−0.500	0.064	0.843	0.157
	MFIA	0.810	0.028	0.270	0.303	0.138	−0.184	0.875	0.125
	TD	0.872	0.004	0.205	0.039	0.337	−0.214	0.963	0.038
	TR	0.883	0.114	0.228	0.126	0.276	−0.156	0.961	0.039
	*K*	0.230	0.473	−0.169	0.529	0.381	−0.128	0.747	0.253
	LD	−0.822	−0.014	−0.302	0.048	−0.255	0.147	0.856	0.144

Late elongation	LAI	0.361	0.460	−0.414	0.435	−0.056	0.245	0.765	0.235
	MFIA	−0.296	−0.182	0.697	0.058	0.456	−0.041	0.820	0.180
	TD	−0.442	−0.362	0.581	−0.336	0.101	−0.429	0.971	0.029
	TR	0.883	0.114	0.228	0.126	0.276	−0.156	0.961	0.039
	*K*	−0.284	−0.160	0.696	0.082	0.464	−0.020	0.813	0.187
	LD	0.321	0.336	−0.405	0.511	0.181	0.460	0.885	0.115

	PH	−0.424	−0.292	0.202	−0.561	0.081	0.052	0.630	0.370
	SNH	−0.364	0.154	−0.684	0.385	0.278	−0.214	0.895	0.105
	SD	0.051	−0.288	0.732	0.117	0.089	0.510	0.903	0.097
	SDW	−0.095	−0.371	0.746	−0.039	0.109	0.502	0.969	0.031
	CY	−0.445	−0.313	0.127	0.385	0.465	0.446	0.875	0.125
	VC	11.342	7.178	5.637	3.025	2.527	1.792		
	%	32.410	20.510	16.100	8.640	7.220	5.120		
	CV %	32.410	52.910	69.020	77.660	84.880	90.000		

Notes: JD: joint degree; SV: special variance; PH: plant height; SNH: stalk number per hectare; SD: stem diameter; SDW: single stem weight; CY: cane yield; VC: variance contribution; CV: cumulative contribution.

**Table 8:** Correlation matrix of main economic traits and canopy trait in sugarcane.

Stages	Variables	PH	SNH	SD	SDW	CY
Tillering	LD	−0.020	0.422	−0.517*	−0.488*	−0.098

Early elongation	LAI	0.292	0.549*	−0.280	−0.165	0.361
	MFIA	−0.386	−0.390	−0.055	−0.192	−0.603*
	TD	−0.258	−0.533*	0.106	0.002	−0.520*
	TR	−0.346	−0.529*	0.154	0.025	−0.498*
	*K*	−0.402	−0.390	−0.019	−0.161	−0.570*
	LD	0.175	0.561*	−0.144	−0.064	0.482*

Rapid elongation	MFIA	−0.535*	−0.248	0.223	0.042	−0.158
	TR	−0.492*	−0.280	0.172	0.004	−0.249
	*K*	−0.490*	0.392	−0.213	−0.354	0.038
	MFIA	0.497*	−0.265	0.471	0.588*	0.395

Late elongation	TD	0.520*	−0.283	0.292	0.436	0.144
	TR	−0.492*	−0.280	0.172	0.004	−0.249
	*K*	0.471	−0.256	0.477	0.587*	0.404

Notes: PH: plant height; SNH: stalk number per hectare; SD: stem diameter; SDW: single stem weight; CY: cane yield. *denotes significant correlation at the level of 0.05.

**Table 9:** Loading matrix of varimax orthogonal rotation factors.

Stages	Characters	Factor 1	Factor 2	Factor 3	Factor 4	Factor 5	Factor 6	JD	SV
Seeding	LAI	0.102	−0.941	0.046	0.196	0.027	−0.051	0.940	0.060
	MFIA	−0.292	0.898	0.126	−0.065	0.012	0.121	0.926	0.074
	TD	0.162	0.928	−0.065	−0.181	−0.183	0.053	0.960	0.040
	TR	0.145	0.946	−0.049	−0.237	−0.056	0.041	0.979	0.022
	*K*	−0.253	0.908	0.091	−0.135	0.003	0.183	0.948	0.052
	LD	−0.351	−0.793	0.212	0.205	0.246	−0.041	0.902	0.098

Tillering	LAI	0.012	0.236	0.104	−0.915	0.184	−0.066	0.942	0.058
	MFIA	−0.228	−0.194	0.357	0.826	0.170	−0.084	0.936	0.064
	TD	−0.130	−0.170	0.021	0.902	0.028	0.286	0.942	0.058
	TR	−0.069	−0.175	0.076	0.967	−0.101	0.016	0.986	0.014
	*K*	−0.201	−0.235	0.254	0.855	0.191	−0.065	0.932	0.068
	LD	−0.021	0.071	0.195	−0.781	0.162	−0.402	0.841	0.159

Early elongation	LAI	−0.451	−0.147	0.807	0.004	−0.149	−0.072	0.904	0.096
	MFIA	0.656	−0.142	−0.618	−0.052	0.199	−0.220	0.923	0.077
	TD	0.515	0.055	−0.802	−0.152	0.145	−0.055	0.959	0.041
	TR	0.553	0.038	−0.792	−0.065	0.177	−0.039	0.970	0.030
	*K*	0.641	−0.137	−0.592	−0.014	0.217	−0.195	0.866	0.134
	LD	−0.454	−0.073	0.816	0.173	−0.096	0.001	0.915	0.085

Rapid elongation	LAI	−0.854	0.171	0.076	0.232	−0.109	−0.118	0.843	0.157
	MFIA	0.858	0.190	−0.259	0.073	0.172	0.029	0.875	0.125
	TD	0.943	0.023	−0.208	−0.172	0.026	0.016	0.963	0.038
	TR	0.934	−0.010	−0.255	−0.063	0.135	0.027	0.961	0.039
	*K*	0.469	−0.147	0.353	0.193	0.569	−0.140	0.747	0.253
	LD	−0.860	0.025	0.300	0.124	0.053	−0.085	0.856	0.144

Late elongation	LAI	0.153	−0.144	−0.126	0.041	0.811	−0.214	0.765	0.235
	MFIA	0.182	0.053	0.386	0.251	−0.414	0.633	0.820	0.180
	TD	−0.055	0.146	0.260	0.192	−0.895	0.204	0.971	0.029
	TR	0.934	−0.010	−0.255	−0.063	0.135	0.027	0.961	0.039
	*K*	0.192	0.044	0.385	0.260	−0.378	0.645	0.813	0.187
	LD	0.151	−0.117	0.009	−0.109	0.913	0.056	0.885	0.115

	PH	−0.358	−0.128	0.085	−0.198	−0.615	0.247	0.630	0.370
	SNH	−0.191	0.028	0.691	−0.093	0.439	−0.423	0.895	0.105
	SD	0.086	0.169	−0.231	0.150	−0.100	0.884	0.903	0.097
	SDW	−0.040	0.139	−0.179	0.084	−0.268	0.915	0.969	0.031
	CY	−0.228	0.211	0.507	−0.073	0.213	0.687	0.875	0.125
	VC	7.668	5.441	5.312	5.275	4.114	3.692		
	%	21.907	37.452	52.628	67.698	79.452	89.999		
	CV %	32.410	52.910	69.020	77.660	84.880			

Notes: JD: joint degree; SV: special variance; PH: plant height; SNH: stalk number per hectare; SD: stem diameter; SDW: single stem weight; CY: cane yield; VC: variance contribution; CV: cumulative contribution.
